# Hydraulic retention time and pH affect the performance and microbial communities of passive bioreactors for treatment of acid mine drainage

**DOI:** 10.1186/s13568-017-0440-z

**Published:** 2017-06-27

**Authors:** Tomo Aoyagi, Takaya Hamai, Tomoyuki Hori, Yuki Sato, Mikio Kobayashi, Yuya Sato, Tomohiro Inaba, Atsushi Ogata, Hiroshi Habe, Takeshi Sakata

**Affiliations:** 1 Environmental Management Research Institute, National Institute of Advanced Industrial Science and Technology (AIST), 16-1 Onogawa, Tsukuba, Ibaraki 305-8569 Japan; 2 Metals Technology Center, Japan Oil, Gas and Metals National Corporation (JOGMEC), 9-3 Furudate, Kosaka-kozan, Kosaka, Akita 017-0202 Japan; 3 Japan Oil, Gas and Metals National Corporation (JOGMEC), 2-10-1 Toranomon, Minato-ku, Tokyo, 105-0001 Japan

**Keywords:** Acid mine drainage, High-throughput sequencing of 16S rRNA genes, Metal removal, Microbial community analysis, Passive treatment

## Abstract

For acceleration of removing toxic metals from acid mine drainage (AMD), the effects of hydraulic retention time (HRT) and pH on the reactor performance and microbial community structure in the depth direction of a laboratory-scale packed-bed bioreactor containing rice bran as waste organic material were investigated. The HRT was shortened stepwise from 25 to 12 h, 8 h, and 6 to 5 h under the neutral condition using AMD neutralized with limestone (pH 6.3), and from 25 to 20 h, 12 h, and 8 to 7 h under the acid condition using AMD (pH 3.0). Under the neutral condition, the bioreactor stably operated up to 6 h HRT, which was shorter than under the acid condition (up to 20 h HRT). During stable sulfate reduction, both the organic matter-remaining condition and the low oxidation–reduction potential condition in lower parts of the reactor were observed. Principal coordinate analysis of Illumina sequencing data of 16S rRNA genes revealed a dynamic transition of the microbial communities at the boundary between stable and unstable operation in response to reductions in HRT. During stable operation under both the neutral and acid conditions, several fermentative operational taxonomic units (OTUs) from the phyla *Firmicutes* and *Bacteroidetes* dominated in lower parts of the bioreactor, suggesting that co-existence of these OTUs might lead to metabolic activation of sulfate-reducing bacteria. In contrast, during unstable operation at shorter HRTs, an OTU from the candidate phylum OP11 were found under both conditions. This study demonstrated that these microorganisms can be used to monitor the treatment of AMD, which suggests stable or deteriorated performance of the system.

**Electronic supplementary material:**

The online version of this article (doi:10.1186/s13568-017-0440-z) contains supplementary material, which is available to authorized users.

## Introduction

Acid mine drainage (AMD) is the major effluent generated from metal and coal mines, and contains a variety of dissolved metals and sulfate. There are various options for treating AMD, such as chemical and biological methods to neutralize AMD and remove harmful metals from the solution (Fu and Wang 2011; Kurniawan et al. 2006). One sustainable method is passive treatment, which relies on the biological, chemical, and physical processes in natural environment systems (Skousen et al. 2017). In the passive treatment of AMD, sulfate reduction is the key reaction. Bioreactors with sulfate-reducing bacteria (SRB) offer advantages such as low operating costs and minimal maintenance costs (Hiibel et al. 2011).

In passive bioreactor systems, SRB reduce sulfate (electron acceptor) in AMD to hydrogen sulfide during oxidation of low molecular carbon sources (electron donor). At neutral pH values, the sulfide is available to precipitate metals as metal sulfides, which are immobilized within the reactor and thus removed from the solution. SRB utilize volatile fatty acids (VFAs; e.g., acetate, propionate, butyrate and lactate) and sugars (e.g., glucose and maltose) as main carbon sources (Hiibel et al. 2011). To provide such low-molecular-weight organic substrates for SRB, local organic wastes such as compost, sawdust, wood chips, and rice straw have been used and are gradually hydrolyzed and fermented to small molecules in the bioreactor (Zagury et al. 2006; Chang et al. 2000). For stable operation of the sulfate-reducing bioreactor, the balance between organic waste degradation and sulfate reduction is critical, which will be determined in part by the hydraulic retention time (HRT) and in part by microbial community structure in the bioreactor. Indeed, the HRT is known to greatly influence the performance of sulfate-reducing bioreactor (Vasquez et al. 2016). To accelerate the treatment, the flow velocity of AMD through the bioreactor should be increased (i.e., HRT should be shortened). In contrast, very short HRT may not allow adequate degradation of rice bran or metabolic activation of SRB, resulting in insufficient metal removal.

Recently, a vertical down-flow laboratory scale packed-bed bioreactor containing rice bran as waste organic material for SRB (designated the Japan Oil, Gas and Metals National Corporation [JOGMEC] process) was constructed, and was operated stably with continuous sulfate reduction and metal removal from AMD (Zn, Cu, and Cd) over the course of one year (Sato et al. 2017). However, in the JOGMEC process, the effects of HRT and influent pH on the reactor performance as well as the microbial community structure degrading rice bran have not been investigated to date.

As for microbial community analysis, the recent development of high-throughput DNA sequencing technologies has enabled analysis of complicated microbial communities in natural environments at high resolution and with high sensitivity (Hori et al. 2014, 2015; Sato et al. 2016). In a sulfate-reducing bioreactor, the seasonal changes in SRB species can be monitored by pyrosequencing (Baldwin et al. 2016); however, little is known about the responses of microbial communities other than SRB to changing conditions during the treatment of AMD. Using a combination of chemical analyses and Illumina sequencing of 16S rRNA genes, the relationship between reactor performance and microbial community structure during operation of the JOGMEC process were analyzed.

## Materials and methods

### Sulfate-reducing bioreactor

The vertical flow sulfate-reducing bioreactor containing rice bran as waste organic material for SRB, the JOGMEC process, was developed (Sato et al. 2017). Briefly, the reactor, comprising a polyvinyl chloride column (1100 mm in height and 250 mm in diameter; approximately 50 l capacity) was set up in our prefabricated laboratory at an abandoned mine site. In addition to the input port on the lid (designated the ‘top’), sampling access ports were also installed at the side of the column at 350, 600, 850, and 1100 mm downflow from the lid (designated port 1, port 2, port 3, and port 4, respectively). The column was first packed with a mixture of 4.7 kg rice husks, 20 kg limestone (3–20 mm in diameter), and 17.5 g field soil as inoculum in an approximately 1000-mm-thick layer. Then, 4.5 kg of rice bran were placed on top of this layer, and the column was sealed and saturated with AMD. With the designed HRT, AMD flowed downwards through a layer of rice bran and then through lower layers consisting of rice husks and limestone. The electron donors for sulfate reduction were provided by the degradation of rice bran in the upper layer of the bioreactor.

### Acid mine drainage (AMD) used in this study

Table 1 shows the characteristics of the original AMD, which was initially passed through an iron-oxidation reactor. The resultant iron concentration became approximately 5 mg l^−1^. In the experiments under the neutral condition, iron-treated AMD neutralized with limestone (pH 6.3) was employed, whereas in the experiments under the acid condition the iron-treated AMD (pH 3.0) was used (Table 1).

**Table 1:** Characterization of original acid mine drainage (AMD) and influent water for the bioreactor

	Original AMD	Influent water^a^	
		Neutral condition	Acid condition
pH	3.53	6.25	3.02
ORP (mV)	361	221	531
Sulfate (mg l^−1^)	320.2	315.9	312.5
Zn (mg l^−1^)	16.29	14.93	16.59
Fe (mg l^−1^)	36.49	1.36	5.90
Cu (mg l^−1^)	6.48	4.21	5.13
Cd (mg l^−1^)	0.070	0.061	0.054
Al (mg l^−1^)	8.55	2.63	7.68
Ca (mg l^−1^)	13.15	71.88	13.77

^a^Original AMD was initially passed through an iron-oxidation reactor, and resultantly, the iron concentration became approximately 5 mg l^−1^. In the neutral condition, iron-treated AMD neutralized with limestone (pH 6.3) was used, while iron-treated AMD (pH 3.0) was used in the acid condition

### The effect of hydraulic retention time (HRT) on process performance and microbial community

The effect of HRT on process performance and microbial community composition was investigated under both the neutral and acid conditions. Continuous flow was started at an HRT of 25 h, and then changed to 12, 8, 6, and 5 h under the neutral condition, and to 20, 12, 8, and 7 h under the acid condition. During the experiment, the reactor was continuously operated without maintenance.

### Sampling and chemical analyses

The HRT reactor samples were obtained from five different sampling ports (i.e., top and ports 1–4) at the end of each HRT, and then the HRT was shortened. The samples were analyzed as follows: pH, oxidation–reduction potential (ORP), and temperature were measured with a LAQUA act D-73 pH/Ion meter (HORIBA Scientific, Japan). The sulfate concentration in sample water was analyzed using a Dionex ICS-2100 ion chromatography system with a Dionex IonPac AS22 column (Thermo Scientific, Japan), as described previously (Sato et al. 2017). The total organic carbon (TOC) concentration of sample water was determined with a total organic carbon analyzer (TOC-L; Shimadzu, Japan). The concentrations of volatile fatty acids (VFAs, i.e., acetate, lactate, butyrate, formate, and propionate), sugars (i.e., glucose, maltose, and lactose), and ethanol in sample water were measured by a high-pressure liquid chromatograph (Alliance e26951; Waters, Japan) equipped with an RSpak KC-811 column (Shodex, Japan), a photodiode array (2998; Waters), and a refractive index detector (2414; Waters). The concentrations of the respective metals (Zn, Cu, and Cd) were determined using an SPS3100 Plasma Spectrometer (Hitachi High-Tech Science, Japan) according to the General Rules for Atomic Emission Spectrometry (JIS K 0116:2014).

### Nucleic acid extraction and amplification of 16S rRNA genes for Illumina sequencing

Fifty bioreactor samples from the five sampling ports were stored at −20 °C as pellets until use. Nucleic acid was extracted from each sample using a direct lysis protocol involving bead beating (Noll et al. 2005). After treatment with RNase (Type II-A; Sigma-Aldrich, MO, USA), we quantified purified DNA using NanoDrop Lite (Thermo Fisher Scientific, MA, USA), which was subsequently used as a template for PCR with a high-fidelity DNA polymerase (Q5; New England Biolabs, MA, USA). Amplification of the V4 region of 16S rRNA genes was performed using the universal primers 515F and 806R, both of which were modified to contain an Illumina adapter region, and the latter of which contained a 12 bp barcode for multiplex sequencing (Caporaso et al. 2012). The PCR thermal profiles were as described previously (Navarro et al. 2015; Sato et al. 2016), except a total of 25–35 PCR cycles was employed. The amplicons were purified first with an AMPure XP Kit (Beckman Coulter, CA, USA) and then using a Wizard SV GEL and PCR Clean-up System (Promega, Japan). The barcode-encoded DNA library and an initial control (PhiX; Illumina, CA, USA) were subjected to paired-end sequencing with a 300-cycle MiSeq Reagent kit (Illumina) on a MiSeq sequencer (Illumina).

### Sequence data processing

The PhiX, low-quality (Q < 30), and chimeric sequences were removed and the paired-end sequences were assembled as described previously (Itoh et al. 2014; Aoyagi et al. 2015a). Briefly, contaminating PhiX sequences in the Illumina sequence libraries were detected using a homology search against the Greengenes database (DeSantis et al. 2006) using the Burrows–Wheeler Aligner version 4.0.5 (Li and Durbin 2009). The paired-end sequences were joined using a fastq-join tool in the ea-utils software package, version 1.1.2-301 (Aronesty 2013). The sequences in each library were characterized phylogenetically using the QIIME software package version 1.7.0 (Caporaso et al. 2010). The operational taxonomic units (OTUs) were grouped using a 97% sequence identity cut-off. Representative sequences for each OTU were assigned with the BLAST program in the NCBI nucleotide sequence database. Using the program QIIME (Caporaso et al. 2010), α-diversity indices (e.g., Chao1, Shannon, and Simpson reciprocal) and the weighted UniFrac distances for principal coordinate analysis (PCoA) were calculated (Hori et al. 2015; Aoyagi et al. 2015b).

### Nucleotide sequence accession numbers

Nucleotide sequences obtained from Illumina sequencing analyses based on 16S rRNA genes have been deposited in the MG-RAST database (http://metagenomics.anl.gov/) under the project title “Microbial dynamics in passive sulfate-reducing bioreactor in 2017” with the ID numbers 4742659.3, 4742666.3, and 4742701.3–4742748.3 (50 libraries).

## Results

### Effects of HRT on the chemical parameters of the bioreactor under the neutral condition

**Fig. 1:**
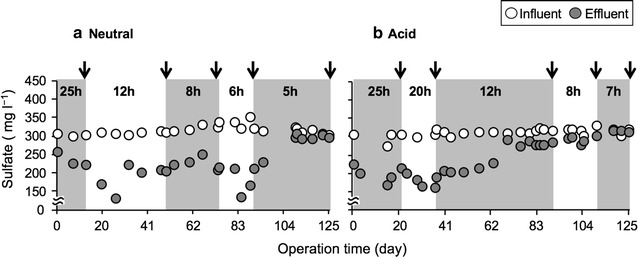
Effect of hydraulic retention time (HRT) on sulfate concentration during 125-day operation under the neutral (**a**) and acid (**b**) conditions. The HRT was changed from 25 to 12 h, 8 h, 6 h, and 5 h under the neutral condition, and to 20 h, 12 h, 8 h, and 7 h under the acid condition. *Symbols* influent, *open circles*; effluent, *gray circles*. *Arrows* indicate the sampling points for depth direction analysis in respective HRTs

The HRT was shortened stepwise from 25 to 5 h under the neutral condition (Fig. 1a). The mean pH values of the effluent were 7.1, and the temperature shifts were shown in Additional file 1: Figure S1A.

Initial sulfate concentrations of the influent were 273–352 mg l^−1^ (average: 313 mg l^−1^). Sulfate reduction apparently occurred throughout the bioreactor in the HRT range from 25 to 6 h, because the sulfate concentrations in the effluents were lower than those in the influents, whereas only a small decrease in sulfate concentration was observed at 5 h HRT (Fig. 1a). Regarding metal removal, the concentrations of dissolved metals (Zn^2+^, Fe^2+^, Cu^2+^ and Cd^2+^) in the effluents were consistently lower than the national effluent standards (Zn, 2 mg l^−1^; Fe, 10 mg l^−1^; Cu, 3 mg l^−1^, and Cd, 0.03 mg l^−1^) (Additional file 1: Figure S1C, E).

**Fig. 2:**
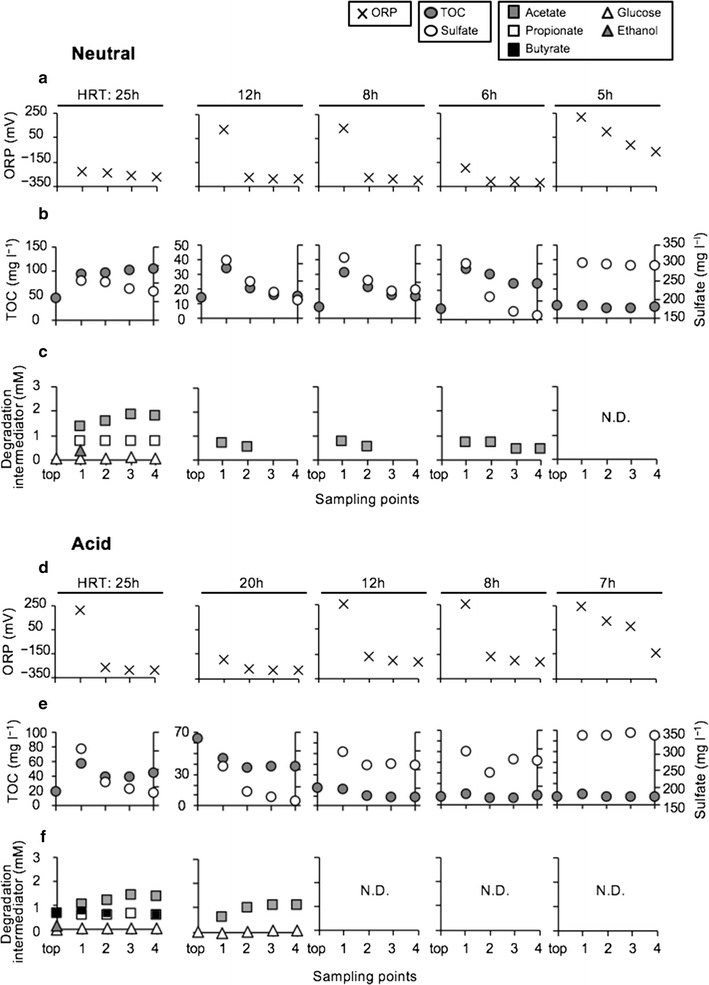
Effects of HRT and pH on oxidation–reduction potential (ORP) (**a**, **d**), total organic carbon (TOC)/sulfate concentrations (**b**, **e**), and low-molecular-weight organic substrate concentrations (**c**, **f**) at each depth of the bioreactor under the neutral (**a**–**c**) and acid (**d**–**f**) conditions. *Symbols* ORP, *saltires*; TOC, *gray circles*; sulfate, *open circles*; acetate, *gray squares*; propionate, *open squares*; butyrate, *closed squares*; glucose, *open triangles*; ethanol, *gray triangles*

Next, the chemical parameters of effluent samples were analyzed. Under the neutral condition, oxidation-reduction potential (ORP) values for the 25 and 6 h HRTs ranged from −200 to −350 mV at sampling ports (Fig. 2a), indicating that the reducing condition was maintained throughout the reactor. At 12 and 8 h HRTs, the ORP values of each port 1 were greater than +50 mV, whereas those of ports 2–4 were approximately −300 mV. This finding suggests that an aerobic atmosphere formed in the upper part but anaerobic conditions prevailed in the lower part of the reactor. In contrast, at a 5 h HRT, ORP values were greater than −100 mV throughout the reactor, including port 4 (Fig. 2a). As a result, under the neutral condition, the ORP values in lower part of the reactor increased at particular HRTs; the low ORP condition suitable for SRB activity was maintained up to a 6 h HRT (Fig. 2a).

Total organic carbon (TOC) and sulfate concentrations varied slightly according to depth at a 25 h HRT, whereas their concentrations decreased in the lower part of the reactor (i.e., from ports 2 to 4) at 12–6 h HRTs (Fig. 2b), suggesting that sulfate reduction was coupled with organic matter oxidation in the bioreactor. At the 25 h HRT, the acetate and propionate concentrations at ports 2–4 were 1.4–1.8 and 0.7–0.8 mM, respectively (Fig. 2c). Glucose was present at concentrations of around 0.1 mM throughout the reactor. At 12 to 6 h HRTs, 0.4–0.8 mM acetate was detected in the lower parts of the reactor. In contrast, these low-molecular-weight organic substrates were not found at a 5 h HRT. After all, under the neutral condition, rice bran digestion and sulfate reduction occurred in the bioreactor at ≤6 h HRTs (Fig. 2b, c).

### Effects of HRT on the chemical parameters of the bioreactor under the acid condition

Under the acid condition, the HRT was shortened stepwise from 20 to 7 h (Fig. 1b). During the operation, the mean pH values of the effluent were 6.9 (Additional file 1: Figure S1B).

Sulfate reduction activity in the bioreactor shifted to be weak during 12 h HRT (Fig. 1b). Although the concentrations of Zn^2+^, Cu^2+^ and Cd^2+^ in the effluents were also consistently lower than the national effluent standards (Additional file 1: Figure S1D, F), the Fe concentration increased to greater than the Japanese standard at an 8 h HRT, reaching 15.2 mg l^−1^ after a 7 h HRT (Additional file 1: Figure S1D).

Under the acid condition, ORP values at port 1 reached >+200 mV for all HRTs (with the exception of the 20 h HRT) (Fig. 2d). At the 20 and 12 h HRTs, the ORP values of ports 2–4 ranged from −280 to −200 mV, however, the values at the 8 and 7 h HRTs were approximately −100 mV, including at port 4 (Fig. 2d). As a result, the low ORP condition suitable for SRB activity was maintained up to a 12 h HRT under the acid condition (Fig. 2d).

In the lower part of the reactor, both TOC and sulfate concentrations decreased at 25 and 20 h HRTs, while no such change was observed after shorter HRTs (i.e., 12–7 h HRTs) (Fig. 2e). The concentrations of acetate and glucose at 25 and 20 h HRTs were 0.7–1.5 and 0.02–0.1 mM, respectively (Fig. 2f). At the 25 h HRT, 0.1–0.7 mM lactate, formate, and butyrate were also detected (butyrate; Fig. 2f, lactate and formate; data not shown).

Therefore, sulfate reduction likely occurred in the lower parts of the reactor, as indicated by the presence of low but detectable levels of acetate (i.e., 0.005–1.5 mM). After all, under the acid condition, both rice bran digestion and sulfate reduction occurred in the bioreactor at ≤20 h HRTs (Fig. 2e, f).

### Effects of HRT on the diversity of microbial communities in the direction of bioreactor depth under the neutral condition

A total of approximately 6.3 million Illumina sequences was obtained from 50 samples under both neutral and acid conditions, corresponding to an average of 126,154 sequences per library (minimum, 45,711; maximum, 186,537; standard deviation, 33,478) (Additional file 1: Table S1). Alpha diversity indices (i.e., Chao1, Shannon, and Simpson reciprocal) of the Illumina sequence data were determined based on an equal number of sequences (*n* = 40,525; Additional file 1: Table S1). These diversity indices revealed few differences between the two conditions.

**Fig. 3:**
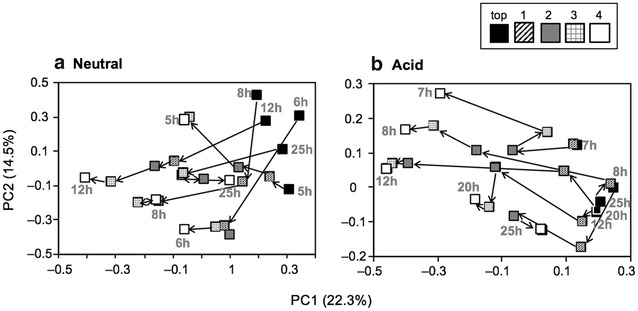
Principal coordinate analysis (PCoA) scatter plot of 16S rRNA sequences based on the weighted UniFrac obtained by Illumina sequencing of the same number of sequences (40,525) of 25 samples from each depth of the bioreactor under the neutral (**a**) and acid (**b**) conditions. The depth of the bioreactor is as follows: *top*, *closed squares*; port 1, *hatched squares*; port 2, *gray squares*; port 3, *plaid squares*; port 4, *open squares*. *Arrows* indicate the direction from *top* to *bottom* of the bioreactor. The *numbers* beside the plots indicate their respective HRTs

Then, community similarities were evaluated by principal coordinate analysis (PCoA) of the Illumina sequence data based on weighted UniFrac distances using an equal number of sequences (*n* = 40,525; Fig. 3). The PCoA scatter plot indicated shifts in the microbial communities according to depth from the top to port 4. The trends were similar among microbial communities during stable operation; i.e., 12–6 h HRTs under the neutral condition (Fig. 3a). The plots at the top were clustered at the upper right. With increasing depth, the plots gradually shifted to the lower left on the panel during stable operation, but shifted to the upper left at the unstable 5 h HRT (Fig. 3a). A considerable change in the community was observed between the 6 h HRT and 5 h HRT. Additionally, under the neutral condition, the distance between the top and port 4 increased during stable operation as compared to that during unstable operation, suggesting that the microbial communities at these reactor positions (i.e., the top and port 4) changed.

**Fig. 4:**
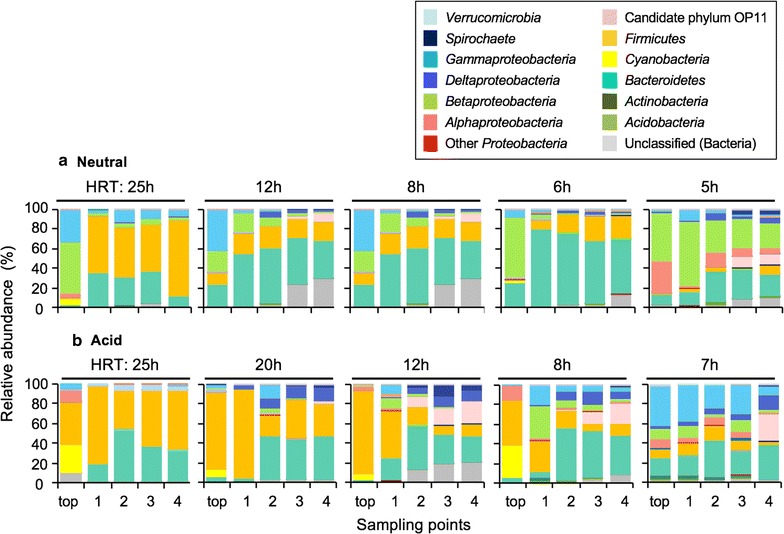
Phylum- and class-level structural changes of the microbial community during HRT operations under the neutral (**a**) and acid (**b**) conditions. Samples obtained at each depth of the reactor (top and ports 1–4) were analyzed by high-throughput sequencing of 16S rRNA genes. The phylogenetic groups are indicated by *colors* and their taxonomies are shown in the *upper section of the graph*. The *color* version of this figure is available online

Phylum- and class-level phylogenetic analyses of Illumina sequence data were conducted using the QIIME software to clarify the changes in microbial community composition according to depth in bioreactors operated at the various HRTs (Fig. 4). The microbial compositions changed at the boundary between stable and unstable HRTs under the neutral condition (i.e., 6 and 5 h HRTs; Fig. 4a), especially at the top and port 4. In the top at a 6 h HRT, the class *Betaproteobacteria* predominated, accounting for 62% of the total population. At a 5 h HRT, the relative abundance of the class *Alphaproteobacteria* increased to 32% of the total. In the port 4 at a 6 h HRT, the phyla *Firmicutes* and *Bacteroidetes* accounted for 23% and 53%, respectively, of the total. At a 5 h HRT, the relative abundance of class *Betaproteobacteria* and the candidate phylum OP11, of which there are no reports of cultured strains, increased to 26% and 11%, respectively.

### Effects of HRT on the diversity of microbial communities in the direction of bioreactor depth under the acid condition

The community similarities were also evaluated by PCoA of the Illumina sequence data under the acid condition, and the PCoA scatter plot indicated shifts in the microbial communities according to depth from the top to port 4. The trends were similar among microbial communities during stable operation; i.e., 25–20 h HRTs under the acid condition (Fig. 3b). The microbial community structure according to depth from the top to port 4 shifted from right to middle during stable operation (i.e., at 25 and 20 h HRTs), while it shifted from right to left during unstable operation (i.e., at 12, 8, and 7 HRTs) (Fig. 3b). A considerable change in the community was observed between the 20 h HRT and 7 h HRT under the acid condition. Additionally, this distance decreased during stable operation, suggesting that the microbial communities between at the top and at the port 4 changed.

Phylum- and class-level phylogenetic analyses of Illumina sequence data revealed the changes in microbial community composition according to depth in bioreactors operated at the various HRTs (Fig. 4b). The microbial compositions changed at the boundary between stable and unstable HRTs under acid condition (i.e., 20 and 7 h HRTs; Fig. 4b), especially at the top and port 4. In the top at a 20 h HRT, the phylum *Firmicutes* predominated, accounting for 79% of the total. At a 7 h HRT, the relative abundances of *Gammaproteobacteria* and *Bacteroidetes* were 41% and 16% of the total, respectively. At port 4, the phyla *Firmicutes* and *Bacteroidetes* dominated at the 20 h HRT, whereas the candidate phylum OP11 became predominant at a 7 h HRT.

### Key microorganisms in AMD treatment under the neutral condition

The operational taxonomic unit (OTU)-level phylogenetic analysis of Illumina sequence data revealed that the microbial community composition of the samples obtained from the top and port 4 differed between 6 and 5 h HRTs under the neutral condition (Table 2).

**Table 2:** Top 5 predominant operational taxonomic units (OTUs) at (a) the top and (b) port 4 of the reactor under the neutral and acid conditions

Condition	HRT (h)	OTU number	Relative abundance (%)	Closest relative species	Sequence similarity (%)	Accession number	Phylum or class
(a)							
Neutral	6	32917	57.94	*Polaromonas hydrogenivorans*	100	KU179860	*Betaproteobacteria*
		23591	21.63	*Flavobacterium johnsoniae*	100	EU984149	*Bacteroidetes*
		90098	6.48	*Pseudomonas extremaustralis*	100	KU508802	*Gammaproteobacteria*
		103770	2.37	*Arthrospira platensis*	86.3	NR125711	*Cyanobacteria*
		21834	1.41	*Variovorax boronicumulans*	96.0	GQ205102	*Betaproteobacteira*
	5	71905	44.71	*Dechloromonas aromatica*	100	NR074748	*Betaproteobacteria*
		87319	31.50	*Novosphingobium sediminicola*	100	KU297717	*Alphaproteobacteria*
		58036	2.19	*Candidatus* Symbiothrix dinenymphae	84.3	AB088917	*Bacteroidetes*
		32917	1.89	*Polaromonas hydrogenivorans*	100	KU179860	*Betaproteobacteria*
		59736	1.81	*Paludibacter propionicigenes*	98.4	AB910740	*Bacteroidetes*
Acid	20	77981	53.63	*Clostridium saccharobutylicum*	100	GU060306	*Firmicutes*
		103770	7.12	*Arthrospira platensis*	86.3	NR125711	*Cyanobacteria*
		16806	6.34	*Clostridium bowmani*	98.0	NR114879	*Firmicutes*
		80315	5.40	*Clostridium akagii*	99.2	NR025352	*Firmicutes*
		68792	3.56	*Bacteroides graminisolvens*	94.1	KT302372	*Bacteroidetes*
	7	90098	37.72	*Pseudomonas extremaustralis*	100	KU508802	*Gammaproteobacteria*
		59736	5.93	*Paludibacter propionicigenes*	98.4	AB910740	*Bacteroidetes*
		61127	5.84	*Microbacter margulisiae*	97.6	NR126216	*Bacteroidetes*
		30357	2.99	*Acidiphilium rubrum*	100	KC924949	*Alphaproteobacteria*
		102362	2.60	*Stenotrophomonas maltophilia*	100	KT825837	*Gammaproteobacteria*
(b)							
Neutral	6	58036	24.35	*Candidatus* Symbiothrix dinenymphae	84.3	AB088917	*Bacteroidetes*
		68792	9.31	*Bacteroides graminisolvens*	94.1	KT302372	*Bacteroidetes*
		16806	8.00	*Clostridium bowmani*	98.0	NR114879	*Firmicutes*
		59736	6.87	*Paludibacter propionicigenes*	98.4	AB910740	*Bacteroidetes*
		26361	5.97	*Trichococcus pasteurii*	100.0	KR857435	*Firmicutes*
	5	58670	11.87	OP11 uncultured bacterium	91.4	UEU68664	Candidate phylum OP11
		64300	9.30	*Thiobacillus thioparus*	100	KC542801	*Betaproteobacteria*
		19080	9.12	*Sediminibacterium goheungense*	100	NR133854	*Bacteroidetes*
		26361	7.73	*Trichococcus pasteurii*	100	KR857435	*Firmicutes*
		96149	6.57	*Bifidobacterium cuniculi*	76.5	LC071801	*Actinobacteria*
Acid	20	59736	16.62	*Paludibacter propionicigenes*	98.4	AB910740	*Bacteroidetes*
		103278	11.15	*Desulfatirhabdium butyrativorans*	94.5	NR043578	*Deltaproteobacteria*
		68792	7.38	*Bacteroides graminisolvens*	94.1	KT302372	*Bacteroidetes*
		16806	5.36	*Clostridium bowmani*	98.0	NR114879	*Firmicutes*
		85528	5.27	*Psychrosinus fermentans*	98.8	NR115860	*Firmicutes*
	7	58670	29.61	OP11 uncultured bacterium	91.4	UEU68664	Candidate phylum OP11
		94556	17.64	*Paludibacter propionicigenes*	98.4	AB910740	*Bacteroidetes*
		90098	6.43	*Pseudomonas extremaustralis*	100	KU508802	*Gammaproteobacteria*
		20362	5.26	*Desulfovibrio mexicanus*	99.2	NR028776	*Deltaproteobacteria*
		31360	3.82	*Paludibacter propionicigenes*	94.5	AB910740	*Bacteroidetes*

The five most dominant OTUs at the top of the reactor under the neutral and acid conditions were extracted from the Illumina sequence library (Table 2a). During stable operation under the neutral condition at a 6 h HRT, OTU 32917 was the predominant species affiliated within the class *Betaproteobacteria*, accounting for 57.9% of the total population. This OTU was related to *Polaromonas hydrogenivorans* (100% sequence similarity). Regarding *Bacteroidetes*, OTU 23591 accounted for 21.6% of the total. This OTU was related to *Flavobacterium johnsoniae* (100% sequence similarity). During unstable operation at a 5 h HRT, within the class *Betaproteobacteria*, OTU 71905, which is related to *Dechloromonas aromatica* (100% sequence similarity), accounted for 44.7% of the total. Concerning the *Alphaproteobacteria*, OTU 87319, which is related to *Novosphingobium sediminicola* (100% sequence similarity), accounted for 31.5% of the total.

Table 2b lists the five most dominant OTUs at port 4 under the neutral and acid conditions. Under the neutral condition at a 6 h HRT within the phylum *Bacteroidetes*, the OTUs 58036 and 68792 predominated, accounting for 24.5 and 9.3% of the total, respectively. The closely related species of these OTUs were *Candidatus* Symbiothrix dinenymphae (84.3% sequence similarity) and *Bacteroides graminisolvents* (94.1% sequence similarity), respectively. Although not listed in Table 2b because of their lower abundance, SRB such as *Desulfosporosinus* spp. were also identified (approximately 2% of the total; data not shown). Low relative abundances of SRB species were observed previously in a passive bioreactor (Baldwin et al. 2016). At a 5 h HRT (unstable operation), the relative abundance of OTU 58670 increased to account for 11.9% of the total. This OTU was related to the predominant candidate phylum OP11 (uncultured bacterium). The OTUs 64300 (*Thiobacillus thioparus*; 100% sequence similarity) and 19080 (*Sediminibacterium goheungense*; 100% sequence similarity), were the next most dominant species.

### Key microorganisms in AMD treatment under the acid conditions

Under the acid condition, the OTU-level phylogenetic analysis of Illumina sequence data between 20 and 7 h HRTs were conducted (Table 2). The five most dominant OTUs at the top of the reactor were extracted (Table 2a). At a 20 h HRT (stable operation), the genus *Clostridium* OTUs 77981, 16806 and 80315 were predominant. The most dominant OTU 77981 (53.6% of the total) had 100% similarity with *C. saccharobutylicum*. At a 7 h HRT (unstable operation) within the class *Gammaproteobacteria*, OTU 90098 was predominant, accounting for 37.7% of the total. This OTU was related to *Pseudomonas extremaustralis* (100% similarity).

Table 2b lists the five most dominant OTUs at port 4. Under the acid condition at port 4, OTU 59736 accounted for 16.6% of the total. This OTU was related to *Paludibacter propionicigens* (98.4% sequence similarity). The sulfate-reducing bacterial OTU 103278, which was related to *Desulfatirhabdium butyrativorans* (94.5% sequence similarity), was the third most dominant species (11.2% of the total). In contrast, at a 7 h HRT (unstable operation), OTU 58670, which is related to the candidate phylum OP11, was the predominant species (29.6% of the total).

## Discussion

In this study, the effects of HRT and pH on the reactor performance and microbial community structure according to depth of the JOGMEC process for the treatment of AMD were investigated. Changes in various chemical parameters (e.g., ORP value, consumption of TOC, and sulfate) under the neutral and acid conditions were monitored to evaluate the treatment of AMD. Illumina sequencing revealed a dynamic transition of the microbial communities at the top and port 4 of the reactor in response to decreasing HRT, as well as the populations of key microorganisms in the reactions.

Under both conditions, both TOC (or acetate) and strictly anaerobic conditions (approximately −300 mV ORP) in the lower part of the bioreactor were necessary for efficient AMD treatment by the JOGMEC process (Fig. 2). The low-molecular-weight organic substrates produced by rice bran degradation at the upper part (top) were provided to the lowest part (port 4), and sulfate reduction occurred throughout the bioreactor, resulting in acceleration of metal removal.

During stable operation; i.e., 12 to 6 h HRTs under the neutral condition and 25 to 20 h HRTs under the acid condition, microbial community structures were similar (Fig. 4); the phyla *Firmicutes* and *Bacteroidetes* predominated under both the neutral and acid conditions. Most genera within the phylum *Firmicutes* were *Clostridium* spp., fermentative organisms that grow on sugars and produce organic acids. Also, the phylum *Bacteroidetes* is a highly efficient degrader of complex carbohydrates essential to the global carbon cycle. Co-existence and accumulation of these two phyla within the bioreactor may be an important indicator of stable operation of AMD treatment by the JOGMEC process.

Comparison between stable and unstable operation; i.e., 6 h and 5 h HRTs under the neutral condition and 20 h and 7 h HRTs under the acid condition, revealed differences in microbial community structures at both the top and port 4 (Figs. 3, 4; Table 2). Aerobic bacteria that can degrade complex organic matter were found at the top port at a 6 h HRT under the neutral condition (Table 2a). *P. hydrogenivorans* is a psychrotolerant bacterium able to utilize a variety of organic acids, alcohols, and some simple sugars (Sizova and Panikov 2007). Also, *F. johnsoniae* can utilize polysaccharides such as chitin and has glycoside hydroxylase family 31 enzymes, which exhibit various substrate specificities (Gozu et al. 2016; Larsbrink et al. 2016). In contrast, a small amount of rice bran was degraded at a 5 h HRT (TOC concentrations were very low [<10 mg l^−1^, Fig. 2b]), likely because this condition might not provide adequate time for growth of the microorganisms that degrade rice bran such as *P. hydrogenivorans* and *F. johnsoniae*. In fact, *Dechloromonas* sp. and *Novosphingobium* sp., predominated at the top port at a 5 h HRT under the neutral condition, both of which are known to be the degrader of hydrocarbons, oil, and aromatic compounds (Salinero et al. 2009; Liu et al. 2005). Under the acid condition (stable operation), anaerobic fermentative *Clostridium* spp. predominated at the top port at a 20 h HRT (Table 2a). Rice bran was degraded, as indicated by detection of appropriate amounts of TOC and acetate in the lowest part of the reactor (Fig. 2e). A longer HRT might facilitate accumulation of anaerobes that can degrade complex carbohydrates.

Compared to the acid condition, a greater diversity of microorganisms participated in degradation of rice bran under the neutral condition. Hence, the neutral condition was more suitable for rice bran-degrading microorganisms at the top of the reactor, and their consumption of oxygen led to a decrease in the ORP value in the lower part of the reactor. Such anaerobic conditions and low-molecular-weight organic substrates promote the metabolic activities of SRB and enhance metal removal. Additionally, the neutral condition would be more likely to precipitate metal sulfide than the acid condition.

In contrast, during unstable operation, i.e., a 5 h HRT under the neutral condition and 12 to 7 h HRTs under the acid condition, the abundance of a common OTU related to candidate phylum OP11 increased (Fig. 4; Table 2). OP11-related organisms are yet-to-be cultured, and have been identified at other metal bioremediation sites (Baldwin et al. 2016). Although the function of the OP11-classified OTU is unknown, its presence may be indicative of unstable operation.

In conclusion, passive AMD treatment by the JOGMEC process was successfully accelerated from 25 h HRT to 6 h HRT under the neutral condition and up to 20 h HRT under the acid condition. To clarify the dynamics of microbial communities associated with changing reactor parameters, high-throughput Illumina sequencing of 16S rRNA genes was employed. Organic waste degraders and fermenters were key microorganisms in AMD treatment in response to changes in both HRT and pH. The phyla *Firmicutes* and *Bacteroidetes* co-existed and predominated throughout the bioreactor, with the exception of the top rice bran layer, leading to metabolic activation of SRB. The accumulation of candidate phylum OP11 species at port 4 was indicative of unstable operation of the system. Detection of these OTUs in the bioreactor may improve our understanding of the JOGMEC process. To support our findings, further investigation and optimization of the process using other AMD samples and soil inocula are now underway.

## Additional file

**Additional file 1: Figure S1.** Effect of HRT on pH/temperature, Zn/Fe concentrations, and Cu/Cd concentrations of the effluent water during 125-day operation under the neutral and acid conditions. **Table S1.** Illumina sequencing data of 16S rRNA genes and the calculated α-diversity indices.

## Abbreviations

AMD: acid mine drainage
SRB: sulfate-reducing bacteria
HRT: hydraulic retention time
ORP: oxidation–reduction potential
TOC: total organic carbon
VFA: volatile fatty acid
OTU: operational taxonomic unit
