# Supplementary material for: Hydraulic retention time and pH affect the performance and microbial communities of passive bioreactors for treatment of acid mine drainage
Source: AMB Express. 2017 Jun 27;7:142. doi: 10.1186/s13568-017-0440-z (PMC5487312; doi:10.1186/s13568-017-0440-z)
Supplement: Supplementary file 1 — Additional file 1: Figure S1. Effect of HRT on pH/temperature, Zn/Fe concentrations, and Cu/Cd concentrations of the effluent water during 125-day operation under the neutral and acid conditions. Table S1. Illumina sequencing data of 16S rRNA genes and the calculated α-diversity indices. [file 13568_2017_440_MOESM1_ESM.pdf]

Supplemental materials

AMB express

ORIGINAL ARTICLE

Hydraulic retention time and pH affect the performance and microbial communities of passive bioreactors for treatment of acid mine drainage

Tomo Aoyagi[1,†] • Takaya Hamai[2,†,*] • Tomoyuki Hori[1,†] • Yuki Sato[2] • Mikio Kobayashi[3] • Yuya Sato[1] • Tomohiro Inaba[1] • Atsushi Ogata[1] • Hiroshi Habe[1,*] • Takeshi Sakata[3]

[1]Environmental Management Research Institute, National Institute of Advanced Industrial Science and Technology (AIST), 16-1 Onogawa, Tsukuba, Ibaraki 305-8569, Japan
[2]Metals Technology Center, Japan Oil, Gas and Metals National Corporation (JOGMEC), 9-3 Furudate, Kosaka-kozan, Kosaka, Akita 017-0202, Japan
[3]Japan Oil, Gas and Metals National Corporation (JOGMEC), 2-10-1 Toranomon, Minato-ku, Tokyo 105-0001, Japan

[†]These authors contributed equally to this work.

*Co-corresponding author
Takaya Hamai
hamai-takaya@jogmec.go.jp
Phone: +81-186-25-8337, Fax: +81-186-29-3849

Hiroshi Habe
hiroshi.habe@aist.go.jp
Phone: +81-29-861-6247, Fax: +81-29-861-8326

Aoyagi *et al.*, Fig. S1

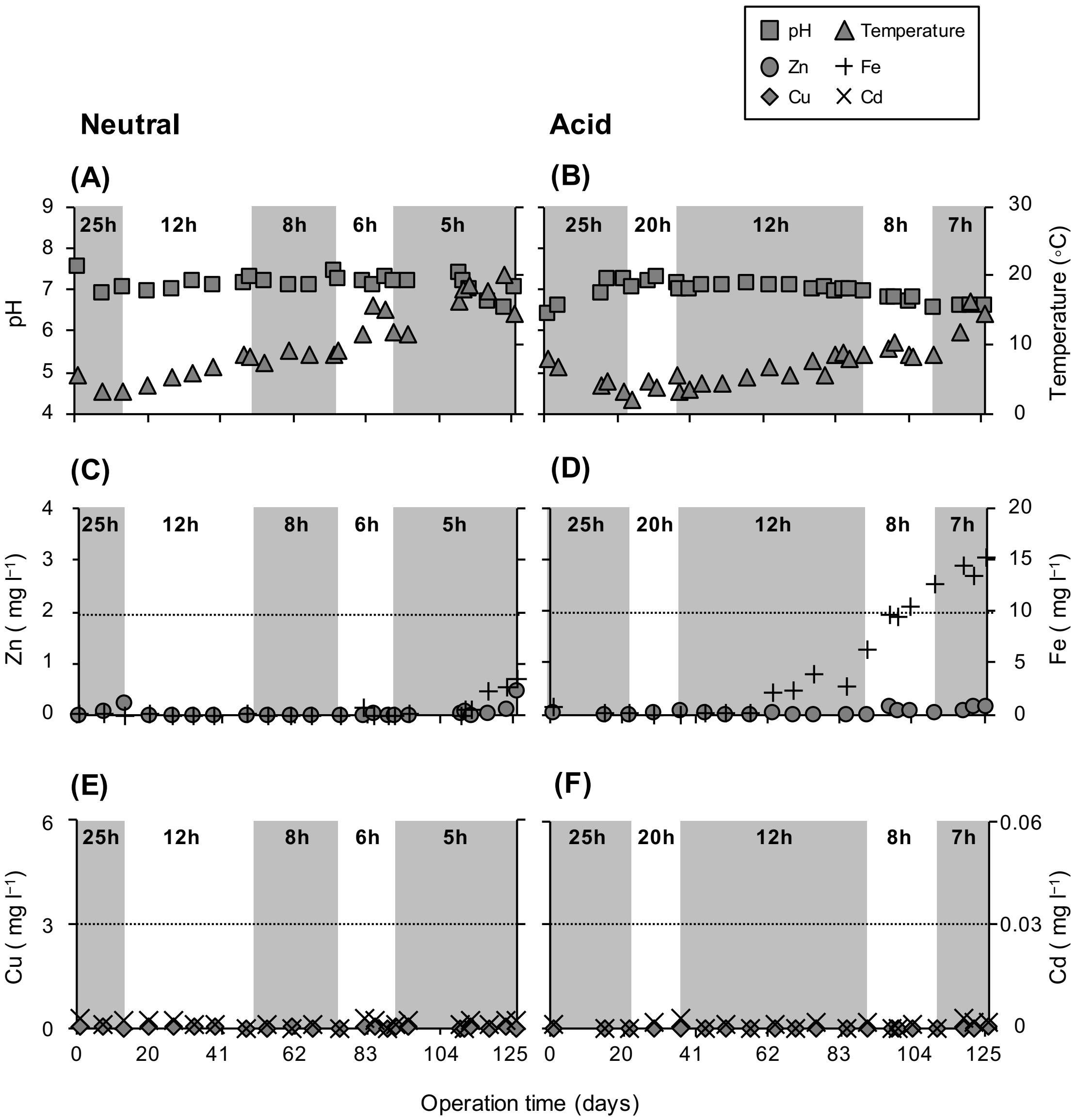

Fig. S1 Effect of HRT on pH/temperature (A, B), Zn/Fe concentrations (C, D), and Cu/Cd concentrations (E, F) of the effluent water during 125-day operation under the neutral (A, C, E) and acid (B, D, F) conditions. Symbols: pH, squares; temperature, triangles; Zn, circles; F, crosses; Cu, diamonds; Cd, saltires. Dotted lines indicate the national effluent standards in Japan.

Table S1 Illumina sequencing data of 16S rRNA genes and the calculated α-diversity indices.

Condition	HRT	Sampling points	No. of sequences	Alpha-diversity [a]		
				Chao1	Shannon	1/Simpson
Neutral	25h	Top	113,515	2577.3 (± 251.6)	2.45 (± 0.018)	2.96 (± 0.012)
		1	141,983	4554.0 (± 281.5)	4.21 (± 0.011)	7.22 (± 0.033)
		2	173,526	6558.4 (± 292.5)	5.28 (± 0.008)	14.11 (± 0.082)
		3	107,351	5797.6 (± 222.7)	5.39 (± 0.017)	12.83 (± 0.097)
		4	122,644	4632.6 (± 193.7)	4.64 (± 0.021)	8.38 (± 0.072)
	12h	Top	154,060	3525.1 (± 260.5)	3.61 (± 0.018)	5.37 (± 0.033)
		1	137,124	4777.3 (± 345.3)	4.26 (± 0.026)	5.22 (± 0.036)
		2	133,501	7503.0 (± 245.0)	4.95 (± 0.019)	5.17 (± 0.045)
		3	147,248	5028.2 (± 273.0)	4.12 (± 0.018)	4.95 (± 0.035)
		4	168,139	3199.1 (± 169.3)	3.58 (± 0.008)	4.86 (± 0.014)
	8h	Top	103,096	6180.9 (± 275.6)	5.53 (± 0.016)	8.28 (± 0.072)
		1	167,919	5704.5 (± 231.1)	4.61 (± 0.012)	8.05 (± 0.058)
		2	137,441	8212.1 (± 359.2)	6.04 (± 0.022)	15.25 (± 0.113)
		3	149,300	9571.8 (± 294.8)	6.17 (± 0.018)	16.23 (± 0.114)
		4	127,902	8684.3 (± 356.3)	5.77 (± 0.017)	13.51 (± 0.096)
	6h	Top	45,711	2937.6 (± 126.8)	3.48 (± 0.009)	2.81 (± 0.008)
		1	110,623	4100.2 (± 158.7)	4.53 (± 0.013)	6.06 (± 0.032)
		2	100,466	3382.7 (± 174.9)	4.28 (± 0.015)	5.04 (± 0.034)
		3	101,865	4712.9 (± 159.6)	4.92 (± 0.014)	8.08 (± 0.061)
		4	77,265	7613.1 (± 193.2)	6.31 (± 0.014)	13.12 (± 0.095)
	5h	Top	92,043	2091.6 (± 121.7)	3.11 (± 0.014)	3.44 (± 0.016)
		1	73,952	5013.0 (± 322.4)	3.87 (± 0.007)	2.77 (± 0.009)
		2	92,403	6848.4 (± 271.4)	6.02 (± 0.011)	14.62 (± 0.103)
		3	84,878	4959.7 (± 166.4)	6.26 (± 0.013)	24.62 (± 0.199)
		4	89,898	5370.8 (± 210.5)	6.12 (± 0.013)	22.71 (± 0.092)

Condition	HRT	Sampling points	No. of sequences	Alpha-diversity [a]		
				Chao1	Shannon	1/Simpson
Acid	25h	Top	122,858	3292.8 (± 256.7)	3.08 (± 0.011)	4.09 (± 0.014)
		1	145,305	4256.3 (± 221.9)	4.74 (± 0.015)	11.11 (± 0.072)
		2	154,368	5138.7 (± 350.3)	5.36 (± 0.015)	14.23 (± 0.099)
		3	147,796	6360.4 (± 413.5)	5.41 (± 0.028)	14.59 (± 0.095)
		4	156,005	5675.9 (± 319.6)	5.59 (± 0.009)	16.85 (± 0.049)
	12h	Top	165,071	4186.1 (± 226.4)	3.67 (± 0.018)	3.49 (± 0.029)
		1	147,817	4622.7 (± 270.8)	4.72 (± 0.012)	8.54 (± 0.043)
		2	178,522	9341.3 (± 266.8)	6.33 (± 0.021)	18.39 (± 0.224)
		3	186,537	8661.0 (± 304.4)	6.01 (± 0.012)	17.55 (± 0.165)
		4	172,193	6598.1 (± 313.2)	5.94 (± 0.017)	17.97 (± 0.207)
	8h	Top	169,871	4345.7 (± 296.9)	4.28 (± 0.013)	6.03 (± 0.043)
		1	114,047	6459.3 (± 374.9)	5.38 (± 0.012)	7.56 (± 0.057)
		2	124,167	4956.2 (± 300.2)	4.77 (± 0.012)	9.73 (± 0.066)
		3	141,275	6178.8 (± 261.8)	5.07 (± 0.021)	11.56 (± 0.088)
		4	166,864	5436.6 (± 237.6)	4.68 (± 0.007)	9.23 (± 0.049)
	6h	Top	92,802	3746.1 (± 188.1)	4.15 (± 0.006)	6.26 (± 0.029)
		1	111,953	5721.1 (± 476.7)	5.61 (± 0.012)	10.87 (± 0.084)
		2	131,552	5340.1 (± 283.8)	5.11 (± 0.023)	8.25 (± 0.076)
		3	159,767	6446.4 (± 231.6)	5.09 (± 0.018)	8.58 (± 0.063)
		4	125,637	5878.4 (± 212.5)	5.03 (± 0.017)	8.79 (± 0.054)
	5h	Top	80,791	9974.7 (± 228.0)	6.44 (± 0.018)	8.43 (± 0.083)
		1	109,869	9207.0 (± 375.4)	6.77 (± 0.011)	11.16 (± 0.084)
		2	79,364	10178.4 (± 377.6)	6.94 (± 0.019)	15.72 (± 0.149)
		3	85,905	10590.0 (± 388.7)	7.18 (± 0.024)	17.68 (± 0.215)
		4	83,521	8013.8 (± 292.5)	5.73 (± 0.017)	9.22 (± 0.061)

a Each diversity index (Chao1, Shannon, and Simpson reciprocal) was calculated based on an equivalent number of sequences (n= 40,525) subsampled from the original libraries. The average data and the standard deviation of each subsample were calculated 10 times for standardization. For each index, higher values represent more diverse microbial communities.
